# Passive Immunization in the Prevention and Treatment of Viral Infections

**DOI:** 10.1002/eji.202451606

**Published:** 2025-05-25

**Authors:** Romila Moirangthem, Yotam Bar‐On

**Affiliations:** ^1^ Department of Immunology Rappaport Faculty of Medicine Technion‐Israel Institute of Technology Haifa Israel

**Keywords:** antibodies, passive immunization, viral immunity, viruses

## Abstract

The basic concepts of passive immunization and the potential of antibody therapy to confer immunity against infectious diseases were introduced already in the late 19th century. This approach was also later implemented to extensively treat and prevent infections, but with the development of effective vaccines, it became restricted to only a few medical conditions such as snake bites, neutralization of toxins, and prevention of rabies infection. This has dramatically changed in the last decade, as antibodies have been widely used in the clinic for the treatment of COVID‐19 and the prevention of respiratory syncytial virus (RSV) infections. A stepping‐stone for the progress in monoclonal antibody generation was the development of single‐cell antibody cloning techniques that made it possible to develop effective neutralizing antibodies against highly mutable viruses such as influenza virus and HIV‐1. Here, we review the use of passive immunotherapy in the clinic for treating and controlling SARS‐CoV‐2 and RSV infections. We further discuss key developments that have made it possible to use monoclonal antibodies against the highly mutable HIV‐1 and influenza virus and advanced clinical trials designed to evaluate the efficacy of such an approach. Finally, we present recent findings that demonstrate that passive immunization can elicit long‐term immunity in the host.

## Graphical abstract

The development of new technologies for generating monoclonal antibodies has made it possible to extend the use of monoclonal antibodies for controlling viral infections. Here, we discuss the advances in antibody cloning techniques and the current use of passive immunization both as a preventative measure and treatment of viral infection.

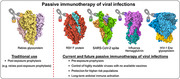

## 1 Introduction

Effective prevention and control of viral infections have been proven to be immensely challenging [1]. This is mainly attributed to the high diversity seen in viruses that are circulating in the human population, as well as the ability of viruses to adapt and evolve in response to antiviral agents [2, 3]. Rapid induction of protective immunity in vaccines might prevent initial infection of the host and by that can limit de novo viral mutations that occur during viral replication. However, breakthrough infections, in which the virus is able to establish an infection in a vaccinated individual, are frequently recorded in highly diverse viruses [4]. The development of a universal vaccine that would elicit broadly neutralizing antibodies (bNAbs) against the vast majority of circulating viral strains could potentially minimize such events, but several hurdles hinder the development of such vaccines [5]. These include the fact that bNAb elicitation requires a combination of sequential immunogens and prolonged coevolution of the antigen with the host B‐cell response [5, 6].

The inability to eradicate highly mutable viruses has underscored the need for effective antiviral therapeutics. Two core principles of antiviral therapy are the use of drug combinations to reduce viral escape and the targeting of conserved viral epitopes to increase the breadth of the therapeutic agent [7, 8]. Similar approaches are already well implemented in cancer therapy, in which a combination of biological drugs or immune checkpoint inhibitors significantly extended the relapse‐free survival of patients in several types of advanced cancers [9, 10, 11]. In the treatment of viral infections, the most profound example of the importance of drug combination is antiretroviral therapy (ART), which is given to HIV‐1‐infected individuals [12]. Remarkably, the three‐drug combination ART that became available in 1996 has transformed HIV‐1 infection from a deadly disease to a manageable chronic disease [13, 14, 15].

With the complexity of targeting viral antigens, antibodies possess several properties that highlight their potential for effective treatment and prevention of viral infections [5, 16, 17]. Monoclonal antibodies bind a particular epitope of the target protein, which is usually well‐defined and characterized by structural studies of the antibody‐antigen complex. This facilitates a rational design of antibody combinations that bind distinct antigenic sites, either on the same viral protein or on different viral proteins in order to limit the emergence of antibody‐resistant viral mutants [18, 19, 20]. Binding and neutralization of distinct viral epitopes could also be achieved by designing bispecific antibodies, which are used as alternatives for antibody combination therapy and can simplify the design of antibody formulation in comparison to a more complex formulation that is required for the administration of antibody cocktail [21, 22, 23]. Moreover, one of the most unique properties of antibodies over other chemical compounds is that antibodies are bifunctional molecules. While the antibody variable Fab mediates the specific binding to the viral antigen, the constant Fc domain interacts with FcγR‐expressing immune cells, which augment and activate the host immune response against both antibody‐bound virions and infected cells [24, 25, 26, 27]. The Fc‐mediated immune activation affects both cellular immune responses by inducing antibody‐dependent cellular cytotoxicity (ADCC) against the virally infected cells or phagocytosis (ADCP) of the infected cells [24, 25]. Additionally, it can improve adaptive immune responses by generating immune complexes that augment antigen uptake and presentation by antigen‐presenting cells [28, 29].

Passive immunization also provides protection for newborn babies against pathogens they might encounter shortly after birth, either by maternal antibodies that are transferred to the embryo through the placenta or by antibodies in breast milk [30]. In 2023, the use of passive immunization against viral infections has reached a new peak, as the administration of monoclonal antibodies with extended half‐life against respiratory syncytial virus (RSV) infection has been approved by the US Food and Drug Administration for use in healthy infants and not only in high‐risk individuals [31, 32]. This is an important milestone for using passive immunization in healthy individuals in efforts to prevent viral infections. In this review, we discuss antibodies and passive immunization strategies that are currently used in the clinic for the prevention and treatment of viral infections or pandemic outbreaks, as well as novel antibodies that are currently being developed and evaluated in clinical trials against highly mutable viruses. Additionally, we review recent seminal findings that illustrate the long‐term effect of passive antibody administration on the host immune response.

## 2 Broadly neutralizing antibodies for treatment and prevention of HIV‐1

Despite 40 years of extensive research, attempts to develop an effective HIV‐1 vaccine and cure for HIV‐1 infection are still ongoing [5, 33–35]. This is an unprecedented challenge in comparison with the historic success of vaccines and antiviral therapeutics in controlling and even eradicating other infectious diseases [1]. The main difficulty is a result of two unique properties of HIV‐1: its ability to establish a state of latent infection and the virus's error‐prone replication [36, 37]. The latter is a consequence of the high error rates of the HIV‐1 reverse transcriptase of about 7.3 × 10^−5^ [38]. The high mutation rate is mostly evident in the high antigenic variability of the HIV‐1 Env protein, the sole target for HIV‐1 antibodies, which is constantly evolving in response to selective pressure exerted by the humoral immune response [39]. The limited success in eliciting antibodies that can recognize a vast majority of circulating Env variants by active immunization is the bottleneck for the development of an effective HIV‐1 vaccine [5]. However, this hurdle has accelerated the attempts to clone such antibodies and administrate them as preventative measures to healthy individuals in areas with a high frequency of HIV‐1 infections, and for treatment of HIV‐1‐infected individuals [19, 40–45].

Despite the high mutation rate of the HIV‐1 Env, several vulnerability sites have been identified as promising targets for Env antibodies that include both epitopes at the CD4‐binding site and outside of receptor binding sites [46]. For example, the b12 antibody that was directed against the Env CD4‐binding site and was shown to neutralize 75% of the primary isolates tested [47], the 2G12 antibody that targets the high‐mannose cluster on the glycan shield of HIV‐1 [48] and a cross‐clade neutralizing antibody against the gp41 domain [49]. However, it was not until Michel C. Nussenzweig and colleagues established a single‐cell antibody cloning method for the isolation and cloning of HIV‐1 bNAb that more potent and broad antibodies were developed [19, 42, 50–53]. This technique has made it possible to thoroughly examine the B‐cell repertoire of HIV‐1 elite neutralizers, in which bNAbs are naturally developed following the extended coevolution of HIV‐1 and the host's B‐cell responses [5, 42, 50]. These second‐generation HIV‐1 bNAbs were able to neutralize a broader panel of HIV‐1 strains and also showed increased neutralization potency [54]. Several of these antibodies have been shown to effectively suppress HIV‐1 viremia in macaque models and human studies, and are currently being tested in advanced clinical trials for HIV‐1 prevention and treatment (Table 1). The most advanced bNAbs include 3BNC117 and VRC01 that target the CD4 binding site on the HIV‐1 envelope, as well as 10–1074 and PGT121 recognize the base of the Env V3 loop and surrounding glycans [42, 50, 53, 55, 56].

**TABLE 1:** Ongoing and initial reports of pivotal trials testing bNAbs for prevention and treatment of HIV‐1.

**Antibody**	**Clinical trial identifier**	**Goal**	**Therapy/Prevention**	**Phase**
3BNC117‐LS,10‐1074‐LS	NCT05245292	To evaluate the safety and antiretroviral activity of the 3BNC117‐LS plus 10‐1074‐LS broadly neutralizing antibody (bNAb) combination plus N‐803, an IL‐15 superagonist complex, in ART‐treated individuals living with HIV during interruption of ART	Therapy	I
3BNC117‐LS, 10‐1074‐LS	NCT04319367 [57]	To investigate the effect of dual long‐acting versions of bNAbs (3BNC117‐LS and 10‐1074‐LS) in a randomized clinical trial powered to answer the question of whether these bNAbs are effective at controlling HIV replication in the absence of ART	Therapy	II
3BNC117‐LS,10‐1074‐LS	NCT06071767	To evaluate the safety, tolerability, and efficacy of therapeutic vaccination with chimpanzee adenovirus (ChAdV)‐ and poxvirus modified vaccinia Ankara (MVA)‐vectored conserved mosaic T‐cell vaccines in a sequential regimen with the Toll‐like receptor 7 (TLR7) agonist vesatolimod (VES) and two broadly neutralizing antibodies (bNAbs)	Therapy	I/IIa
3BNC117‐LS, 10‐1074‐LS	NCT04173819 [58]	To evaluate safety and pharmacokinetics of the Combination Broadly Neutralizing Antibodies, 3BNC117‐LS‐J and 10‐1074‐LS‐J, in Healthy American and African Adults	Prevention	I/II
VRC01	NCT02568215 [59]	To evaluate the safety and efficacy of the human monoclonal antibody (mAb) VRC‐HIVMAB060‐00‐AB (VRC01) in preventing HIV‐1 infection in high‐risk, HIV‐uninfected women	Prevention	IIb
PGT121, VRC07‐523LS, PGDM1400	NCT03721510 [60]	To evaluate the safety, tolerability, pharmacokinetics, and anti‐viral activity of PGT121, VRC07‐523LS, and PGDM1400 for HIV prevention and therapy	Therapy + Prevention	I/IIa

**FIGURE 1:**
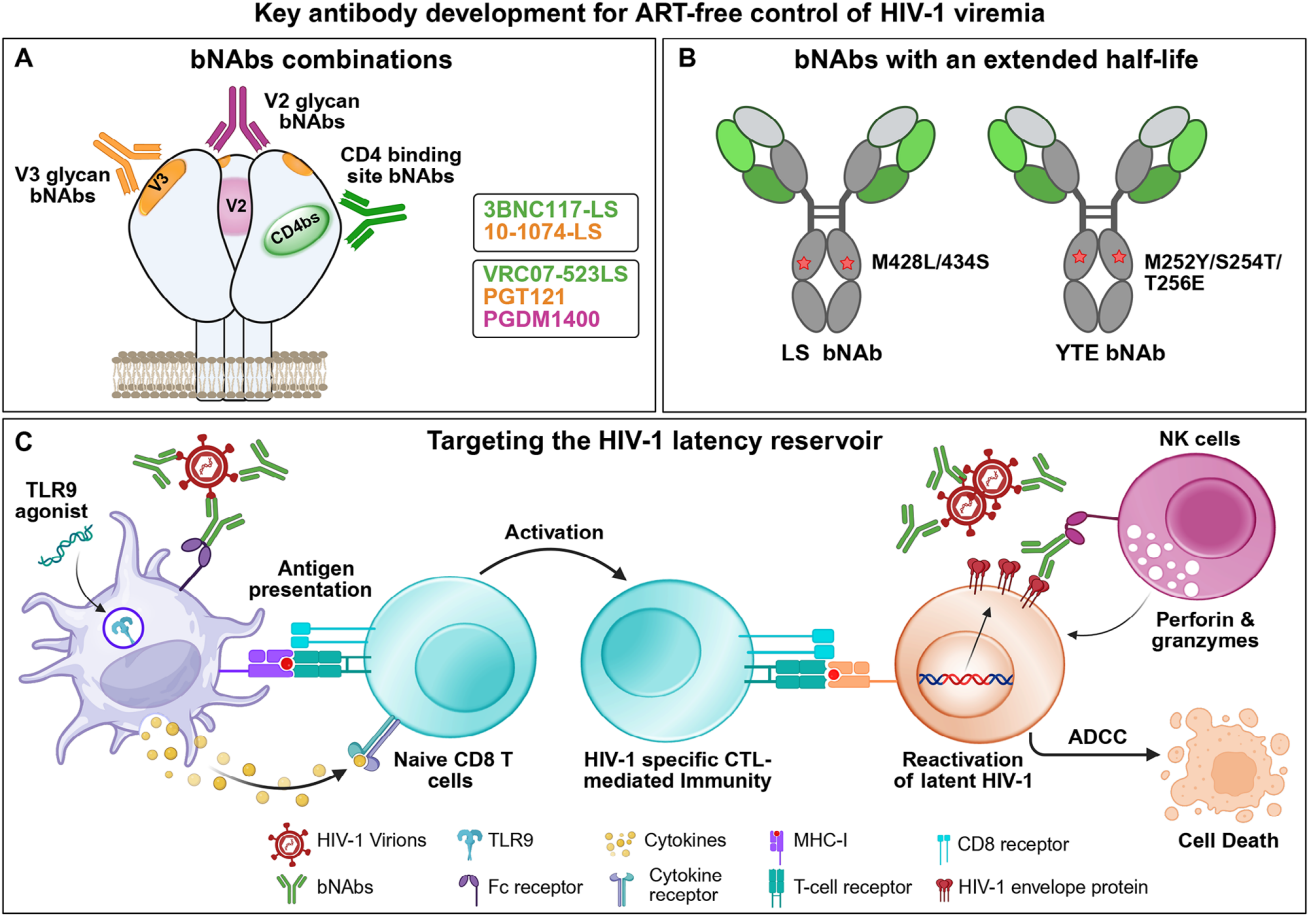
Key antibody developments for ART‐free control of HIV‐1 viremia. (A) The use of bNAbs combinations. The HIV‐1 envelope trimer, with major epitopes targeted by the bNAbs combination in ongoing clinical trials, is shown. The epitope regions are highlighted as follows: the CD4 binding site in green, the V2 apex in magenta, and the V3 glycan supersite in orange. The bNAbs cocktails currently in clinical trials are listed in the grey box, with each bNAb color‐coded according to the specific target site it addresses in the study. (B) Modification of the antibody Fc domain for extended antibody half‐life. Depiction of Fc‐engineered bNAbs. LS and YTE mutants are Fc‐engineered antibodies with mutations (M428L/N434S for LS, M252Y/S254T/T256E for YTE) that significantly extend the half‐life of bNAbs. (C) Novel methods to target the HIV‐1 latent reservoir with bNAbs. A conceptual overview demonstrating the combined effects of Toll‐like receptor (TLR) agonists and bNAbs. TLR agonists and bNAbs‐antigen complexes engage plasmacytoid dendritic cells (pDCs), leading to enhanced viral antigen presentation to naïve CD8^+^ T cells and to increased cytokine release for activation of these cells. This process enhances the stimulation of HIV‐1 specific CTL mediated immunity. Additionally, TLR agonists activate the innate immune system by triggering pDCs, which in turn increases the proportion of activated cytotoxic NK cells. These primed NK cells bind to bNAb‐antigen complexes via Fcγ receptors, inducing antibody‐dependent cellular cytotoxicity (ADCC) to kill HIV‐1 infected CD4+ T cells. Finally, TLR agonists might increase the reactivation of the latent HIV‐1 provirus by cytokine release or cell‐to‐cell‐mediated activation.

After initial human clinical trials indicated that these antibodies are safe and well tolerated [41], a series of studies [19, 29, 42, 52, 53, 55, 61] have examined their ability to effectively suppress HIV‐1 in infected individuals (Figure 1). As current drug‐based therapy can effectively suppress HIV‐1 viremia, it is important to emphasize several advantages of antibody therapy for HIV‐1 infections in comparison to ART. First, the average half‐life of bNAbs is around 21 days, and this can be further extended up to fourfold by the addition of the LS mutation in the antibody Fc domain [62]. This mutation increases the antibody affinity for the neonatal Fc receptor (FcRn) in acidic environments. As the FcRn binding to IgG at low pH is essential for preventing lysosomal degradation of the antibody and for recycling it back into circulation, this Fc modification significantly increases the IgG half‐life [62]. Thus, as the half‐life limitations of ART demand daily administration of the antiviral medications, bNAbs therapy can overcome this difficulty as antibodies can be infused into the HIV‐1 infected individuals only every few months. This is a significant advantage, as poor adherence to ART is one of the leading causes of the increased morbidity of infected individuals and the establishment of new HIV‐1 infections [63]. Another fundamental advantage of antibody‐based therapy for HIV‐1 infected individuals is the ability of antibodies to engage immune cells. Several studies demonstrated that the binding of antibodies to Env that is expressed on the surface of infected CD4^+^ T cells can induce ADCC and the elimination of HIV‐1‐infected cells [24, 64, 65]. This was found to largely contribute to bNAbs anti‐HIV‐1 activity, in addition to the neutralization capacity of these antibodies [64]. Of note, recent studies demonstrated that in addition to the well‐studied Fc effector functions of bNAbs, these antibodies can elicit long‐term cellular and humoral immune responses against HIV‐1 [28, 66, 67]. Thus, unlike ART, bNAbs administration can provide HIV‐1 immunity in the host even after the antibodies are completely cleared from the plasma [28]. Moreover, single‐genome sequencing analysis of HIV‐1 from bNAb‐treated individuals revealed that the emergence of antibody‐resistant HIV‐1 strains is largely limited by the administration of combination antibody therapy that targets two distinct Env epitopes [19].

Another noteworthy limitation of HIV‐1 therapy is the latent HIV‐1 that persists mainly in CD4^+^ T cells as a long‐lived HIV‐1 reservoir [68]. This latent reservoir is resistant to ART, and even decades of treatment do not decrease the latent reservoir of inducible, replication‐competent HIV‐1 [69]. As the HIV‐1 latent reservoir is currently the major obstacle to HIV‐1 cure, testing if bNAbs treatment can reduce or alter the HIV‐1 latent reservoir is a crucial question for future HIV‐1 curative strategies (Figure 1C). This is a complex question given that under normal conditions, the latently infected cells do not express the Env protein, which is the direct target of HIV‐1 bNAbs. Gunst D. Jesper et al. have thoroughly tested the possible impact of bNAbs therapy on the HIV‐1 latent reservoir in a randomized phase 2a TITAN trial in which individuals with HIV‐1 and on ART were administrated with bNAbs with or without a TLR9 agonist. In this study, there were no added benefits for combining the TLR9 agonist with bNAbs. However, antibody treatment alone led to a significant delay in viral rebound upon ART interruption [70]. Additionally, the frequency of CD4^+^ T cells harboring intact proviruses expanded after ART interruption in non‐bNAb recipients but that bNAb administration prevented such expansion at early time points [70]. In a study by Gaebler et al. HIV‐infected individuals received seven doses of a combination of two broadly neutralizing antibodies over 20 weeks in the presence or absence of ART. Two of the individuals from this cohort who received the bNAbs maintained HIV‐1 suppression for one year. Of note, changes in the size and composition of the intact proviral reservoir were also observed in the bNAb‐treated individuals [71]. Thus, this study stresses the need for further investigating the possible use of bNAbs administration for achieving durable antiretroviral therapy‐free control of HIV‐1 viremia in infected individuals (Figure 1).

## 3 Development of Broadly Neutralizing Antibodies for Treatment of Influenza Virus Infection

While the unique properties of HIV‐1 make this virus extremely difficult to suppress, the difficulty in controlling highly mutable viruses is also evident in influenza virus infections, where the diversity of influenza viruses driven by continuous genetic alterations and its widespread, pose a major challenge [72]. Currently, vaccination plays an important role in preventing influenza infections despite a suboptimal protective efficacy, which ranges approximately from 20% to 60%. Moreover, vulnerable populations such as infants, the elderly, and immunocompromised individuals typically respond poorly to the vaccines and are at a higher risk of contracting influenza [73].

bNAbs have emerged as promising candidates for both therapeutic and prophylactic passive immunization against the influenza virus [74]. Several bNAbs targeting influenza A viruses (IAVs) and influenza B viruses (IBVs) have been developed and evaluated in preclinical and clinical studies, demonstrating protective efficacy [74]. The vast majority of the antibodies are isolated from the sera of vaccinated healthy individuals or recovered patients and are elicited against the two influenza virus surface glycoproteins hemagglutinin (HA) and neuraminidase (NA) [74]. The hemagglutinin (HA) homotrimer is made up of two chains, HA1 and HA2, linked by a disulfide bond. HA is typically divided into an immunodominant head region and an immune‐subdominant stem region. The HA head contains the receptor‐binding site (RBS), which is the major target for monoclonal antibodies [75]. These antibodies are highly effective in blocking viral attachment or preventing receptor‐mediated endocytosis, neutralizing the virus, and preventing infection. However, these antibodies are usually strain‐specific and may be ineffective against drifted strains [74, 75]. These problems were addressed by Ian Wilson and colleagues, who isolated bNAbs such as S139/1, C05, and F045‐092 that target the conserved region of the RBS sites [76, 77, 78]. Moreover, the identification of a conserved epitope hidden at the HA head trimer interface has led to the development of novel bNAbs, such as S5V2‐29, H2214, and Flu‐20, that show broad reactivity to most IAV subtypes by inhibiting cell‐to‐cell spread of the virus [79, 80].

**FIGURE 2:**
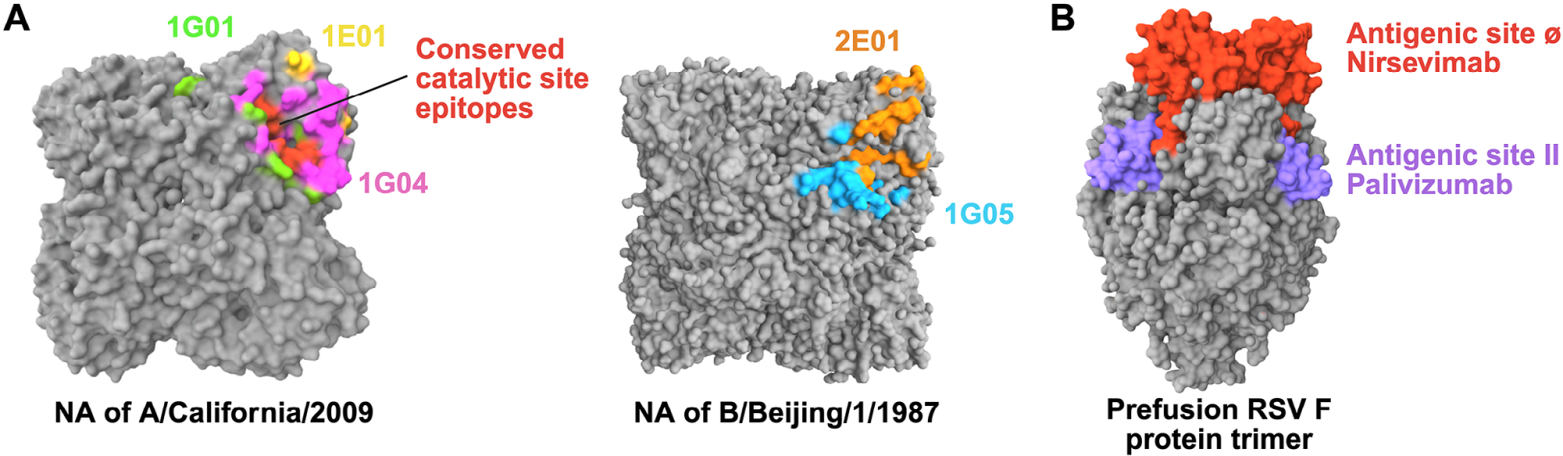
Monoclonal antibodies against influenza NA glycoprotein and RSV F glycoprotein. (A, Left) NA protein from H1N1 subtype A/California/04/2009 in grey (PDB ID: 6Q23) showing the epitope of 1G01 Fab in green, 1E01 Fab in yellow, 1G04 Fab in pink, and common conserved active site epitopes between the antibodies are highlighted in red. (A, Right) NA protein from Influenza B/Beijing/1/87 in grey (PDB ID: 1NSC), in complex with 1G05 Fab in light blue and 2E01 Fab in Orange. (B) The RSV prefusion F trimer in grey with surface epitopes of nirsevimab at antigenic site Ø (red) and palivizumab at antigenic site II (purple). The 3D structures are created using the MolStar graphic tool.

As opposed to the HA head, the slower mutation rate of the HA stem makes it possible for antibodies that target the stem to neutralize multiple IAV subtypes [81]. HA Stem antibodies have been shown to impair viral and endosomal membrane fusion, inhibit viral release, and disrupt HA maturation [81]. They are classified into four types based on their neutralizing breadth: group 1‐specific, group 2‐specific, group 1 and 2 heterosubtypic, and IAV and IBV heterosubtypic. Group 1 HAs mAb such as C179, CR6261, F10, 6F12, 3.1, 3E1, S9‐3‐37 and 222‐1C06 bind to the epitopes in the HA central stalk [82, 83, 84, 85, 86], while the group 2 HAs mAbs like CR8020, CR8043, 9H10 or 12D1, 042‐100809‐2F04 and 41–5E04, bind to the membrane‐proximal base of the HA stem [87, 88]. Anti‐stem heterosubtypic neutralizing antibodies capable of neutralizing both group‐1 and group‐2 influenza strains are rare. However, a few such broadly neutralizing antibodies, such as F16v3, CR9114, 05‐2G02, 81.39a, 39.29/MHAA4549A, MEDI8852, VIS410, and CT149, have been isolated by Antonio Lanzavecchia's group and others [89, 90, 91]. Interestingly, the influenza virus NA has emerged in recent years as a novel key target for influenza virus antibodies. Several previous studies have shown that preexisting NA‐reactive antibodies in humans can reduce infection rates and lessen disease severity, suggesting that NA‐targeting antibodies may be a promising therapeutic option, especially given NA's slower antigenic drift compared with HA [92]. Major progress in targeting this viral protein came with the development of advanced techniques to stabilize the NA tetramer [93] and with the isolation of NA bNAbs by Stadlbaue et al. [94]. These antibodies (1G05, 2E01, 1G04, 1E01, and 1G01) bind to the conserved catalytic site of NA and are shown to be cross‐reactive against both IAVs and IBVs [94] (Figure 2A).

Numerous bNAbs have progressed into clinical trials to assess their safety and therapeutic potential. While the preclinical trials of Crucell's HA stem antibody CR6261 demonstrated promising prophylactic and therapeutic efficacy, in a subsequent randomized, double‐blind, placebo‐controlled phase‐II trial (NCT02371668), where 50 mg/kg of CR6261 was administered to healthy volunteers 24 h after H1N1 infection, no significant prophylactic or therapeutic effects were observed [95]. Moreover, antibody MHAA4549A binds to the conserved stalk helix A, neutralized H1 and H3 subtypes, and was shown to be more effective than oseltamivir in mice and ferrets infected with H1N1 and H3N2, and also showed significant therapeutic efficacy in a human H3N2 challenge model [96]. However, in outpatient trials for uncomplicated influenza A virus, MHAA4549A had a limited impact on the time of viral load clearance compared with the placebo, despite some benefits in symptom resolution and virus replication [97]. MEDI8852 is a broad‐spectrum antibody with strong neutralization against 18 IAV HA subtypes [98]. A Phase 2a study conducted in adults with uncomplicated influenza (NCT02603952) found no significant difference between MEDI8852 alone or in combination with oseltamivir [98]. Thus, it is probable that a combination of HA antibodies that target distinct sites of the HA or antibody cocktail that targets both the influenza virus HA and NA would be a key to the success of antibody‐mediated control of influenza virus infections.

## 4 Antibodies for Controlling the COVID‐19 Pandemic

The outbreak of the COVID‐19 pandemic in late 2019 has emphasized the importance of developing effective anti‐viral antibodies against viruses with a pandemic potential. During this outbreak, the Emergency Use Authorization (EUA) of nine anti‐SARS‐CoV‐2 antibodies provided an alternative therapeutic and preventative strategy until an effective COVID‐19 vaccine was developed and COVID‐19 became an endemic disease [99]. This included casirivimab and imdevimab (Regeneron Pharmaceuticals), bamlanivimab and bebtelovimab (Eli Lilly and Company), sotrovimab (GlaxoSmithKline), regdanvimab (Celltrion) and cilgavimab/tixagevimab (Astra Zeneca) [99, 100]. These antibodies showed effective antiviral activity and potent neutralization of SARS‐CoV‐2, but the emergence of SARS‐CoV‐2 variants of concern (VOCs) such as Omicron (B.1.1.529), BA.2, BA.3, BA.4, and BA.5 significantly impaired the therapeutic capacity of these SARS‐CoV‐2 antibodies [99, 101]. This has initiated the pursuit of developing anti‐SARS‐CoV‐2 antibodies with increased breadth, which will provide effective protection against most circulating variants.

The antibody cocktail of casirivimab and imdevimab (REGEN‐COV) has been one of the most widely used therapeutic antibodies during the COVID‐19 outbreak [102]. They were originally developed as part of an intensive effort in which various potent antibodies were generated from humanized mice and from B cells that were isolated from convalescent humans [103]. REGEN‐COV antibodies are fully human‐neutralizing antibodies that target the receptor binding domain (RBD) on the SARS‐CoV‐2 at distinct, nonoverlapping epitopes [102]. As seen with other viruses [19], the use of antibody combination was designed to provide better coverage against circulating viral variants and limit the emergence of antibody‐resistant strains. Initial in vitro neutralization studies have demonstrated that REGEN‐COV retains its efficacy against emerging variants such as D614G and UK B.1.1.7 [99, 100]. These findings were further supported by large‐scale analysis of SARS‐CoV‐2 spike sequences derived from clinical trials that indicated that the combination of antibodies in the REGEN‐COV was able to limit the emergence of SARS‐CoV‐2 escape mutants [102]. Importantly, the impressive efficacy of REGEN‐COV in reducing COVID‐19‐related hospitalizations or death that was shown in phase‐3 clinical trials [104], was also indicated in a retrospective cohort study aimed to determine the real‐world effectiveness of REGEN‐COV [105]. This study, which included patients infected with the Delta variant, demonstrated a 93.5% treatment efficacy of REGEN‐COV in preventing COVID‐19 death in the 28 days after treatment [105].

At later stages of the pandemic, the continuous evaluation of circulating SARS‐CoV‐2 has hindered the therapeutic ability of monoclonal antibodies to control infection [106, 107]. This was predominantly seen with the spread of the Omicron variant that carried a large number of spike mutations [106]. In vitro analysis of the Omicron variant demonstrated that most of the clinically approved antibodies have reduced neutralization against this variant [106]. Consequentially, in January 2022, the FDA revised the authorizations for monoclonal antibody treatments in the U.S. and limited their usage to cases of infection with SARS‐CoV‐2 variants that were shown to be neutralized by the antibodies [99, 100]. Later, broadly neutralizing monoclonal antibodies that recognize RBD epitopes that are conserved among SARS‐CoV‐2 variants were developed [108] and are likely to play a major role in controlling future COVID‐19 outbreaks.

## 5 Passive Immunization for Protection of Infants from RSV Infections

Respiratory syncytial virus (RSV) is a highly prevalent cause of respiratory infection that can cause severe illness especially in infants and in elderly persons [109]. In the last two years, and after decades of extensive research with many hurdles to overcome, both active and passive immunization strategies for protection from RSV infection have been clinically available [110, 111]. This marks an outstanding milestone for controlling a highly transmissible viral infection through a combination of active immunization and neutralizing antibody administration. RSV is an enveloped single‐stranded, negative‐sense RNA virus that expresses two surface glycoproteins: the G protein and the fusion (F) protein. Development of RSV therapeutics and protective RSV vaccines is focused on targeting these two glycoproteins as they are the only RSV proteins accessible for antibody neutralization [112, 113]. Targeting the RSV F protein has been proven to be a more effective therapeutic approach since it is more conserved than the G protein between different RSV strains [114]. Additionally, several studies have indicated that while the F protein is essential for RSV infection of its target cells, infection can still occur in the absence of the G protein or following its neutralization [115]. This was further supported by the recent study of Griffiths et al. [116] has uncovered the mechanism of RSV entry into cells, in which the RSV‐F glycoprotein interacts with the insulin‐like growth factor‐1 receptor to initiate infection of airway epithelial cells.

The long pursuit of the development of an RSV vaccine has been hindered by several setbacks. The first inactivated RSV vaccine led to an enhanced respiratory disease following natural infection, with an extremely high percentage of hospitalization cases and two RSV‐related deaths [110]. This was later attributed to the low capacity of this vaccine to elicit neutralizing antibodies, which has been proven to be the best correlate for vaccine efficacy [110]. Structural studies of the RSV F glycoprotein have later presented the second challenge in RSV immunization and also provided a mechanistic explanation for the low levels of neutralizing antibodies that were elicited with the inactivated RSV vaccine. These studies revealed that the RSV F protein trimers are dynamic structures and that the prefusion conformation is highly unstable [110, 111, 117]. Moreover, it was demonstrated that the most potent neutralizing antibodies are directed against the antigenic sites Ø and V that are present in the prefusion conformation [110]. Thus, stabilization of the prefusion F conformation was found to favor the elicitation of neutralizing antibodies, and the generation of prefusion‐stabilized F proteins has been a stepping‐stone toward the FDA approval of two RSV vaccines for the elderly population in 2023 [111, 113]. These vaccines, Arexvy and Abrysvo, are currently used to prevent RSV disease in adults 60 years old and above [118].

For infants, who are the other RSV high‐risk population, preventative measurements are highly dependent on passive immunization (Figure 2B). In July 2023, the FDA approved the monoclonal antibody nirsevimab (Beyfortus) for the prevention of RSV lower respiratory tract disease in all infants and in a subgroup of 2‐year‐old children who remain at high risk for serious disease [110, 118]. This is a key landmark in the clinical use of monoclonal antibodies, as nirsevimab is given as a preventive measure for healthy individuals. The long‐term clinical success of nirsevimab could accelerate the use of monoclonal antibodies for pre‐exposure prophylaxis for other viruses such as HIV‐1, where there is no effective vaccine, but very potent monoclonal antibodies were developed [5]. Of note, for infants at the highest risk for RSV lower respiratory tract disease due to preterm labor, chronic lung disease, or congenital heart disease, a humanized RSV monoclonal antibody named palivizumab has been a standard of care for RSV prevention since 1998 [110]. However, nirsevimab has demonstrated an improved neutralization potency in comparison with palivizumab and an extended serum half‐life. The latter is a result of a three amino acid modification (tyrosine, threonine, and glutamate) that was introduced to the nirsevimab Fc region [119, 120, 121]. This Fc modification increases the antibody Fc region affinity to neonatal Fc receptor (FcRn) and therefore prolongs its serum persistence [120]. Thus, a single dose of nirsevimab administration can effectively prevent RSV‐related hospitalizations for 5 months or longer [119, 120, 121].

Currently, one of the most intriguing questions is the capability of RSV to establish a breakthrough infection, a term that describes RSV infection in infants who were administrated with nirsevimab. A study by Fourati et al. [122] analyzed 260 full‐length RSV genome sequences from nirsevimab‐treated breakthrough infections that occurred in France during the 2023–24 RSV season. In accordance with results obtained in nirsevimab late‐phase clinical trials [32], low levels of nirsevimab‐related resistance mutations were observed. No resistance mutations in the nirsevimab binding site were recorded in all the isolated RSV‐A sequences, and only 8% of the RSV‐B sequences carried these mutations [122]. This is an encouraging result for the future clinical efficacy of nirsevimab in preventing RSV infections in infants. However, this also stresses the importance of studying in‐depth how the F‐protein mutations that are far removed from the nirsevimab binding site could still affect its binding or neutralizing capacity. Of note, antibody escape that is mediated by mutations outside of the antibody binding site was previously reported in other viral surface proteins [65].

### 5.1 The Vaccinal Effect of Antibody Administration

The short‐term functions of antibodies are well‐defined and are classified by the different antibody domains that facilitate these functions. The antibody specificity and affinity are determined by the variable fragment (Fab) [123]. The Fab region is also responsible for antigen neutralization, which has been shown to be crucial for the efficacy of vaccines to protect from viral infections [124, 125]. The antibody constant fragment (Fc) mediates downstream effector functions by binding to immune cells through Fc receptors [24]. During viral infection, this interaction facilitates the direct killing of the infected cells by natural killer cells, enhances their phagocytosis by macrophages, and enables better antigen uptake by antigen‐presenting cells [24, 126]. The ability of antibodies to neutralize the virus together with its capacity to induce effector functions can significantly impact the ability of the host to control and clear viral infections. However, thorough analysis of antibody therapy in preclinical and clinical studies has uncovered long‐term immune responses that are elicited during antibody therapy but can augment the host immunity even after complete clearance of the administrated antibody [28, 29, 66, 67].

The discovery of these antibody‐mediated long‐term immune responses, which were later termed vaccinal effects, was made possible due to studies that investigated the cancer patient's immune response in monoclonal antibody‐treated individuals [127]. Initial observations that supported a possible vaccinal effect of antibody therapy were seen in cancer patients who were treated with rituximab, a chimeric IgG1 monoclonal antibody that specifically targets the CD20 and is widely used in the treatment of B‐cell non‐Hodgkin lymphoma [127]. Strikingly, in these individuals, the peak host responses to rituximab therapy may take several months, implying that the antibody's short‐term activity cannot fully explain the efficacy of rituximab treatment [128]. Additional studies have demonstrated augmented cellular and humoral immune responses in antibody‐treated cancer patients, such as in the treatment of HER‐2+ breast cancer [129, 130]. Analysis of HER‐2+ breast cancer patients who were treated with the monoclonal antibody trastuzumab has revealed that the antibody treatment has increased the elicitation of endogenous anti‐HER‐2 antibodies and has also increased the anti‐HER‐2 CD4 T‐cell responses [129, 130]. Preclinical studies by DiLillo and Ravetch [127] that were done in a humanized mice model later uncovered the mechanism by which antibodies can mediate such antitumor vaccinal effects. In these studies, they have demonstrated that the binding of the antibody Fc domain to the Fc receptor on dendritic cells (DCs) can augment antigen presentation by DCs, which in turn will enhance the cellular T‐cell responses [127].

The discovery of antibody vaccinal effect in cancer therapy has prompted the question of whether similar long‐term immune activation also occurs during the antibody therapy of viral infections, as in the course of viral infection, the kinetic of the antibody‐antigen interaction is more transient than in antibodies that target tumor antigens [28]. Clear evidence for antibody‐mediated long‐term cellular immune activation has been demonstrated in HIV‐1 infection. Two seminal studies that followed T‐cell responses in rhesus macaques that were infected with simian immunodeficiency virus (SIV)/HIV chimeric virus (SHIV) and were treated with a combination of bNAbs have demonstrated that the antibodies administration has induced durable control of viremia [66, 67]. Strikingly, in these infected rhesus macaques, early bNAbs treatment (3 days after infection) led to prolonged suppression of viral replication with undetectable viremia during nearly a 6‐year follow‐up [66, 67]. The notion that the antibody therapy has elicited a long‐term immune control of SHIV was supported not only by the extended time of viral suppression that far exceeded the antibody plasma half‐life but also by the rebound viremia that was observed shortly after the depletion of CD8^+^ T cells in the treated macaques [66, 67]. Interestingly, injections of the same antibody combination to viremic HIV‐1‐infected individuals have led to prolonged suppression of viremia, but viral rebound did occur upon termination of treatment [19]. One possible explanation for the different treatment outcomes in humans in comparison with the macaque data is that intrahost HIV‐1 diversity in the infected individuals hindered effective CD8^+^ T cell responses and long‐term viremia control. Nevertheless, a study that examined HIV‐1‐specific CD8^+^ T cells in HIV‐1‐infected individuals who are treated with ART or with bNAb has shown an increase in the frequencies of Pol‐ and Gag‐specific CD8+ T cells in the antibody‐treated individuals [131]. Another study that analyzed the elicitation of antibody responses in HIV‐1 infected individuals following administration of bNAb has uncovered that such a treatment can also affect the host humoral response, where a single injection of bNAb accelerated heterologous neutralizing activity in almost all of the tested individuals [29].

**FIGURE 3:**
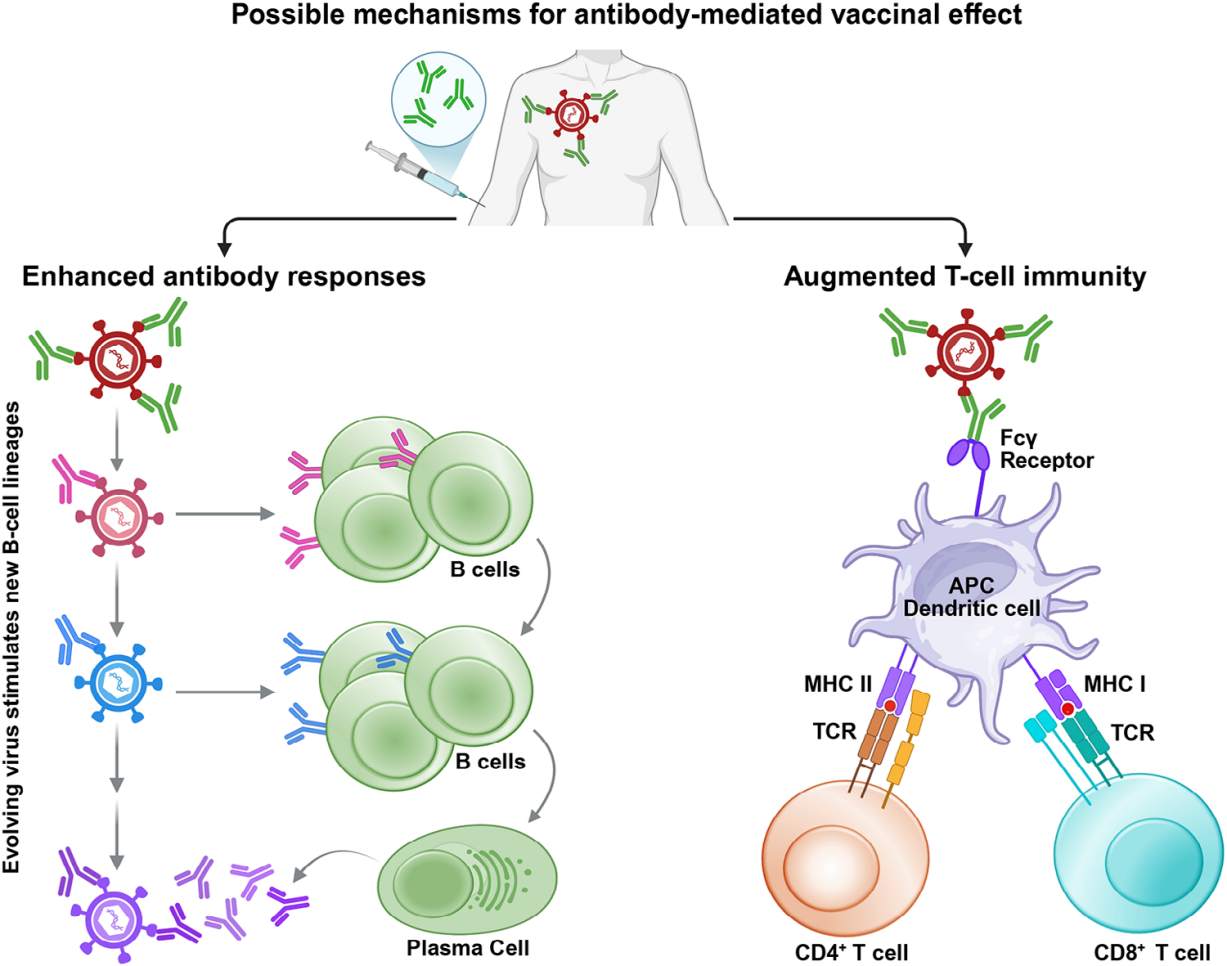
Possible mechanisms for antibody‐mediated vaccinal effect. On the right, immune complexes formed by infused antibodies and circulating viruses act as strong immunogens to interact with Fcγ receptors on dendritic cells promoting phagolysosome fusion, maturation, and increased antigen uptake. This enhances antigen processing and presentation to CD8^+^ and CD4^+^ T cells and strengthens cellular antiviral responses. Alternatively (on the left), the administrated antibody could lead to an enhanced humoral response by inducing antigenic changes in the targeted virus. These viral antigenic changes will lead to the stimulation of new B‐cell lineages and the elicitation of antibodies with improved neutralization capacity.

The fact that passive immunization can change the profile of the elicited antibodies in HIV‐1‐infected individuals could be mediated by antigenic changes in HIV‐1 that occur in response to the injected antibody (Figure 3). Induction of cellular immune responses in these individuals is likely to be mediated by enhanced antigen presentation, as was observed in antibody‐treated cancer patients (Figure 3). This could be the result of the antibody binding to viral particles, which leads to the formation of immune complexes, that can augment antigen uptake and presentation by antigen‐presenting cells due to the binding of the antibody Fc domain to surface Fc receptors [132]. Accordingly, it was shown that modifications in the antibody Fc region, which increase the affinity to specific Fc receptors that are expressed on antigen‐presenting cells, increase antiviral CD8^+^ T‐cell responses in influenza virus‐infected humanized mice [132].

While most studies have focused on the long‐term effects of passive immunization on the adaptive immune responses, alterations in the innate immune response during the antibody administration can dictate and regulate these responses [132]. This was demonstrated by introducing Fc modifications that enable selective binding to the activating Fcγ receptor FcγRIIa, which resulted in increased maturation of dendritic cells [132]. Further studies should evaluate the long‐term effects of passive immunization on other innate immune cells. Alterations in the natural killer (NK) cells landscape are particularly intriguing as these cells express the Fcγ receptor FcγRIIIa which can be engaged by the antibody Fc domain [24]. Moreover, although NK cells are part of the innate immune response, memory NK cells that are capable of eliciting antigen‐specific recall responses were also identified [133]. Finally, long‐term changes in both the function and the frequencies of the NK cell population have been recorded in response to viral infections and other physiological conditions [134, 135].

## 6 Conclusion

The development of bNAbs has promoted the use of passive immunization for the treatment and prevention of viral infections that are caused by highly mutable viruses. Such a therapeutic strategy can protect from viruses such as HIV‐1, where the development of an effective vaccine has been proven to be immensely challenging. Alternatively, it can be used as prophylaxis against a specific virus in high‐risk populations. This has reached its climax with the 2023 FDA approval of the monoclonal antibody nirsevimab (Beyfortus) for the prevention of RSV lower respiratory tract disease in all infants. Another important use of monoclonal antibodies in infectious diseases is in controlling pandemic outbreaks, as was highly evident in the recent COVID‐19 pandemic where antibody therapy provided an effective therapeutic option until effective SARS‐CoV‐2 vaccines were developed. The fact that antibodies are usually well tolerated and have a long half‐life which could be further extended by Fc modifications, has placed them as a favorable therapeutic agent. Interestingly, there are currently many efforts to provide other antiviral drugs with extended plasma half‐life that is similar to the antibody half‐life. For example, the recently developed anti‐HIV‐1 drug, lenacapavir, with a half‐life of up to 12 weeks has been shown to reduce HIV‐1 infections to zero in an efficacy trial in African adolescent girls and young women [136, 137]. A possible advantage of using antibodies for similar purposes, as opposed to these extended half‐life drugs, is the vaccinal effect reported in antibody‐treated individuals and observed in animal models. Whether the vaccinal effect of passive immunization will be successfully translated into drug‐free viremia control in chronic viral infection is still unknown. This is highly dependent on future studies that will provide an in‐depth understanding of the mechanisms that control this long‐term immune activation and the development of antibody modifications that will enhance and augment these responses.

## Author Contributions

Romila Moirangthem and Yotam Bar‐On contributed equally to all aspects of this study.

## Conflicts of Interest

The authors declare no conflicts of interest.

### Peer Review

The peer review history for this article is available at https://publons.com/publon/10.1002/eji.202451606.

## Data Availability

The review manuscript does not contain shared data.
